# Introduction of African Swine Fever into the European Union through Illegal Importation of Pork and Pork Products

**DOI:** 10.1371/journal.pone.0061104

**Published:** 2013-04-15

**Authors:** Solenne Costard, Bryony Anne Jones, Beatriz Martínez-López, Lina Mur, Ana de la Torre, Marta Martínez, Fernando Sánchez-Vizcaíno, Jose-Manuel Sánchez-Vizcaíno, Dirk Udo Pfeiffer, Barbara Wieland

**Affiliations:** 1 Veterinary Epidemiology & Public Health Group, Royal Veterinary College, Hatfield, Hertfordshire, United Kingdom; 2 VISAVET Center and Animal Health Department, Veterinary School, Complutense University of Madrid, Madrid, Spain; 3 Animal Health Research Center, Madrid, Spain; 4 IREC (CSIC-UCLM-JCCM), Ciudad Real, Spain; 5 Swiss Agency for Development and Cooperation, Ulaanbaatar, Mongolia; Institute for Animal Health, United Kingdom

## Abstract

Transboundary animal diseases can have very severe socio-economic impacts when introduced into new regions. The history of disease incursions into the European Union suggests that initial outbreaks were often initiated by illegal importation of meat and derived products. The European Union would benefit from decision-support tools to evaluate the risk of disease introduction caused by illegal imports in order to inform its surveillance strategy. However, due to the difficulty in quantifying illegal movements of animal products, very few studies of this type have been conducted. Using African swine fever as an example, this work presents a novel risk assessment framework for disease introduction into the European Union through illegal importation of meat and products. It uses a semi-quantitative approach based on factors that likely influence the likelihood of release of contaminated smuggled meat and products, and subsequent exposure of the susceptible population. The results suggest that the European Union is at non-negligible risk of African swine fever introduction through illegal importation of pork and products. On a relative risk scale with six categories from negligible to very high, five European Union countries were estimated at high (France, Germany, Italy and United Kingdom) or moderate (Spain) risk of African swine fever release, five countries were at high risk of exposure if African swine fever were released (France, Italy, Poland, Romania and Spain) and ten countries had a moderate exposure risk (Austria, Bulgaria, Germany, Greece, Hungary, Latvia, Lithuania, Portugal, Sweden and United Kingdom). The approach presented here and results obtained for African swine fever provide a basis for the enhancement of risk-based surveillance systems and disease prevention programmes in the European Union.

## Introduction

Transboundary animal diseases (TADs) are diseases with a high potential for spread between countries and of economic, trade and food safety/security importance [1]. Therefore, disease-free countries have developed surveillance programmes to prevent or reduce the risk of disease introduction. Nevertheless, several countries have reported outbreaks of exotic diseases in the past few years [2], and illegal imports have often been shown or suspected to be the cause of primary outbreaks of TADs in countries. In this paper, illegal import(ation) refers to prohibited commodities that are brought in from a foreign country either for personal consumption or for use in trade.

A review by Pharo [3] indicates that foot-and-mouth disease (FMD) incursions into Taiwan in 1997 and United Kingdom in 1967 and 2001 were suspected of being caused by smuggling of meat or meat products. Similarly, the outbreaks of classical swine fever (CSF) in the early 2000 s in several countries of the European Union (EU) were most likely due to illegal imports of pork or pork products followed by illegal feeding of swill to pigs [4], [5]. African swine fever (ASF) is another example of a TAD which has repeatedly been introduced into free areas via illegal imports of pork products by tourists or for commercial purposes, or via the illegal disposal of waste from ships or planes originating from ASF-affected areas. These sources of infection were suspected to be the cause of outbreaks in Spain in the 1960 s [6], in Cuba and Brazil in the 1970 s [7], [8], and more recently in Mauritius in 2007 [9] and the Caucasus in 2007 and 2008 [10], [11].

The EU has experienced outbreaks of exotic diseases in the last few years [2]. One of the reasons for these exotic disease incursions may be the illegal importation of animals and animal products from TADs-infected countries, some of them sharing borders with the EU. In this context, the EU has defined an Animal Health Strategy [12] with an emphasis on disease prevention and enhancement of risk-based surveillance systems. Risk analysis and simulation modelling are increasingly conducted to assess the risk of disease introduction into countries and inform surveillance strategies. However, despite the recognised risk associated with illegal importation of meat and products for the introduction of diseases, very few studies have evaluated such risk. The few published studies that have explicitly considered illegal imports of meat and derived products to assess the risk of disease introduction highlighted that the main constraint is the difficulty of quantifying illegal movements of animals and animal products [13]–[15]. Consequently, these studies mainly used expert opinion both to define the different pathways leading to the release of the disease into a country and exposure of the domestic susceptible population, and to generate estimates for their corresponding probabilities of occurrence. For country-specific risk assessments [13], [14], data on customs seizures was sought to evaluate illegal importation of meat and derived products. However, the availability of such data is rare and, usually, restricted to relatively small areas or a few countries.

ASF is a TAD and an OIE notifiable viral pig disease with high potential for within and between country spread, and for which control solely relies on drastic and costly sanitary measures due to the absence of a vaccine or treatment. ASF is present in most countries of Sub-Saharan Africa, in Sardinia, Transcaucasia and the Russian Federation (RF). The presence of the disease in the RF constitutes an important threat to the EU as highlighted by a number of studies [16]–[20]; proximity and price differences in live animals and animal products between the RF and the EU may promote illegal importation of pork and pork products. The outbreak reported in Ukraine at the time of writing [31st July 2012, 2] further highlights the risk of spread of ASF to the EU.

The objective of this study was to develop a semi-quantitative risk assessment framework for the introduction of a disease into EU through illegal importation of meat and products, based on readily available data and using ASF as an example. Specifically, the model aimed to estimate the risks of release of African swine fever virus (ASFV) into the EU via illegal importation of pork and pork products and subsequent exposure of the domestic pig population, and to allow the comparison of levels of risk between EU member states (MS).

## Materials and Methods

A release and exposure assessment model was developed to assess the risk of introduction of ASFV into the EU through the illegal importation of pork and products, using the same structure and sources of data for all MS so that levels of risk could be compared between countries.

In the absence of readily available data on illegal imports, a semi-quantitative model was developed in Excel 2007 (Microsoft Corp, Redmond, WA) on the basis of information available in the literature, publicly-available data, and expert opinion when no other source of information was identified. The risk was assessed indirectly using proxy indicators (PI), that is factors that were likely to influence the risk, and for which data available at the EU scale were collected in a consistent and regular manner.

**Figure 1:**
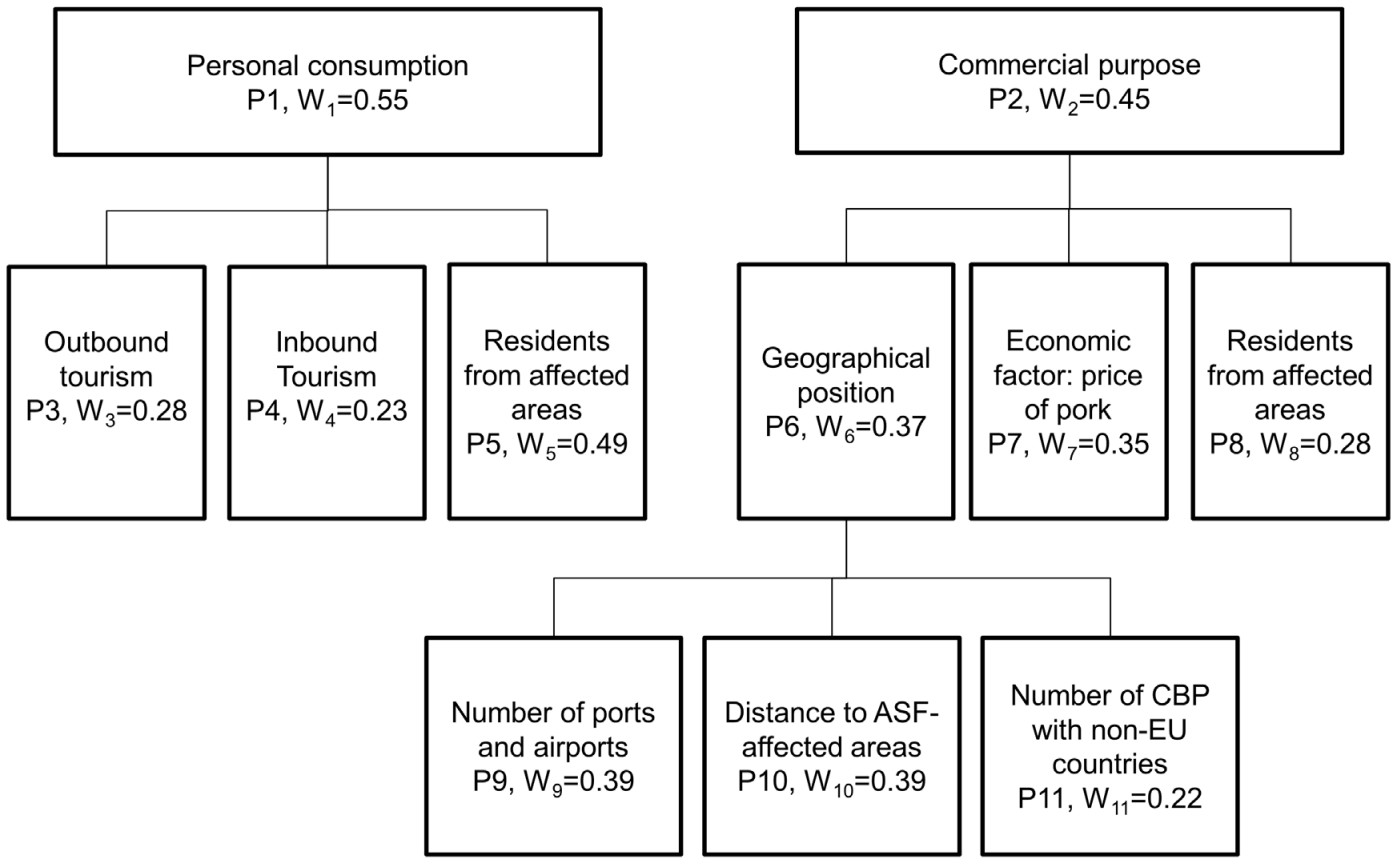
Structure of the release assessment model. Proxy indicators and their relative weights defined for the assessment of the release of ASF into EU via illegal importations of pork and pork products.

**Figure 2:**
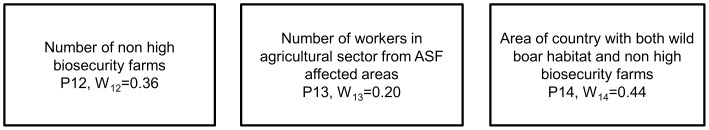
Structure of the exposure assessment model. Proxy indicators defined and their relative weights for the assessment of the exposure of ASF into the European Union via illegal importations of pork and pork products.

Existing risk assessments on illegal imports and current knowledge on the epidemiology of ASF were used to identify factors influencing release and exposure of ASF through illegal imports of pork and products. A total of 14 PI, 11 for release (P1–P11) and 3 for exposure (P12–P14) were identified (Figures 1 and 2). The release of ASF was assumed to occur via illegal imports for both personal consumption and commercial purposes (restaurants, markets, etc.), and PIs were further differentiated according to their relevance for either of these two mechanisms of introduction of pork and pork products (Tables 1 and 2 and Figure 1). The PIs for the exposure assessment were identified assuming that once release via illegal imports occurred, the disease could be transmitted to the domestic pig population via swill feeding, people acting as fomites, or contact with scavenger animals (including wild boar) having access to waste or landfill [14] (Table 3 and Figure 2).

**Table 1:** Description of the proxy indicators included in the risk assessment model for ASF release into the EU through illegal importation of pork and pork products for personal consumption.

Proxy indicator*	Hypothesized relationship with the risk of ASF introduction via illegal import of pork and pork products
**Outbound tourism to ASF affected areas**	It was expected that the risk of release increases with outbound tourism to ASF affected areas. This is because people living in an EU country and travelling to affected areas may bring back ASFV contaminated pork products on their return for their own consumption or as gifts for relatives/friends
**Inbound tourism from ASF affected areas**	It was assumed that the risk of release increases with inbound tourism from ASF affected areas. People from affected areas and travelling to an EU country may bring contaminated pork products with them, for their own consumption during their trip
**EU residents from ASF affected areas**	It was hypothesized that the higher the number of residents from ASF affected areas, the higher the risk of release. The reason for this is that these people may bring contaminated pork products with them on their return from visits to their countries

* proxy indicators are factors likely to influence the risk being assessed.

**Table 2:** Description of the proxy indicators included in the risk assessment model for ASF release into the EU through illegal importation of pork and pork products for commercial purpose.

Proxy indicator*		Hypothesized relationship with the risk of ASF introduction via illegal import of pork and pork products
**Price of pork**		It was assumed that the higher the price of pork in an EU country, the higher the incentive to import meat illegally (as the prospect of benefits increases)
**EU residents from ASF affected areas**		Foreign residents of an EU country were assumed to constitute a clientele for products from their countries of origin. Residents coming from ASF affected areas were hence assumed to be consumers of pork and pork products from their countries, and thus contribute to their risk of illegal imports
**Geographical factors**	Distance to the closest ASF affected area	The countries close to ASF affected areas were assumed at higher risk of release by imports for commercial purpose, as the transport of pork and pork products to these places would be shorter
	Number of ports and airports	The risk of release was assumed to increase with the number of entry points (airports, ports, and road and rail crossings on land borders) into a country
	Number of cross-border points with non EU countries	The risk of release was assumed to increase with the number of entry points (airports, ports, and road and rail crossings on land borders) into a country

* proxy indicators are factors likely to influence the risk being assessed.

**Table 3:** Description of the proxy indicators included in the risk assessment model for exposure of the EU domestic pig population to ASF following its release through illegal importation of pork and pork products.

Proxy indicator*	Hypothesized relationship with the risk of ASF introduction via illegal import of pork and pork products
**Number of workers in livestock sector from ASF affected areas**	People may expose domestic pigs to ASFV by feeding of waste to pets or backyard livestock or by acting as fomites, and staff in pig farms are considered at higher risk of doing so. Residents from ASF affected areas were considered more likely to obtain and consume illegally imported pork and pork products contaminated with ASFV. Hence, the proportion of workers in the agricultural sector from ASF affected areas is expected to increase the risk of exposure of domestic pigs in the case of illegal imports of pork and pork products
**Number of non-high-biosecurity farms**	It was hypothesised that the risk of exposure increased with decreasing farm biosecurity. This is because the following events are considered more likely to occur in non-high-biosecurity farms: swill feeding, contact with people acting as fomites or with scavenger animals having access to waste/landfill and contaminated with ASFV
**Area of country with both wild boar habitat and non-high-biosecurity farms**	It was assumed that the risk of exposure increased with the area of the country covered with both wild boar habitat and low biosecurity farms. In areas with wild boars, low biosecurity farms are at higher risk of contact with wild boars having had access to domestic waste or landfill, and that are either infected or acting as fomites

* proxy indicators are factors likely to influence the risk being assessed.

Each of the PIs was weighted to reflect how much it is considered to influence the risk. The risk of release and exposure of ASF through illegal importations of pork and pork products was then calculated for each MS by weighted linear combination of the PIs’ values and weights.

### Estimation of Proxy Indicators’ Weights

The weights of the PIs contributing to risk of release and exposure were derived from expert opinion, using pair-wise comparison. This elicitation technique is based on the analytical hierarchy process [21], [22], and is typically used to generate preferences or relative weights, which are necessary “when intangible factors need to be added and multiplied among themselves and with tangible factors” [21] i.e. in the present study for the weighted linear combination of the PIs’ values and weights. In addition to the relatively easy implementation and derivation of relative weights (even in the presence of multiple criteria to consider), an important advantage of this elicitation method is that it allows the consistency of elicited weights to be checked. In contrast, other common elicitation methods such as Delphi are typically used to generate quantitative estimates on absolute scales or probabilities (e.g. length of incubation period of the disease under consideration, concentration of the biological hazard in the commodities of interest, probability of contact between herds, etc.) and do not allow for easy assessment of the internal consistency of experts’ answers.

During the elicitation process, a total of 24 scientists involved in an EU project on ASF – ASFRISK (EC, FP7-KBBE-2007-1, Project #211691) initially reviewed the structure of the model and refined the list of PIs. They then provided their opinion on the relative contribution of the PIs to the risk considered, using pair-wise comparison as developed by Saaty [22].

For each level of PIs in Figures 1 and 2, a PI was compared with every other PI (e.g. illegal imports for personal consumption were compared with illegal imports for commercial purpose; or distance to ASF affected countries, number of ports and airports and number of cross-border points with non-EU countries were compared with each other). Experts were asked to assign a preference statement for each PI, based on its relative importance in influencing the risk of release or exposure when compared with the other PIs of the same level and accordingly values were then assigned to each PI [23] (Table 4). The resulting matrix was reciprocal, so that the pair-wise comparison (*a*) for PI *y* and PI *z* was *a_yz_ = a_yz_^−1^*, and all of its diagonal elements were unity, so that *a_yz_ = 1* when *y = z*.

**Table 4:** Values and corresponding preference statements used in the pairwise comparison (Malczewski J (1999) GIS and Multicriteria Decision Analysis. New York, Chichester: John Wiley & Sons. 408 p.).

Description of preference statement	Corresponding value
**Equally important**	1
**Moderately more important**	3
**Strongly more important**	5
**Very strongly more important**	7
**Extremely more important**	9

The pair-wise values provided by each individual expert (*a_yz_*) were used to derive a weight for each PI (*w_y_*) by taking the principal eigenvector of the pair-wise comparisons:
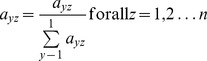




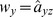
 for all *y = 1, 2…n*

Then, the overall weight for each PI was calculated as the mean of the individual weights given by experts. The overall weights of PIs added up to 1 for each level of PIs.

### Obtaining Data for Proxy Indicators

For each of the release PIs, P3–5 and P7–11 (Figure 1), and for each of the parameters from which the exposure PIs, P12–14 were derived (Figure 2), internet searches were conducted to obtain publicly available datasets collected in a consistent way for all EU MS. Details of the PIs, source of data, assumptions and uncertainty of the data are shown in Tables S1 and S2. For several of the release PIs and most of the exposure PIs, Eurostat [24] provided the most complete data set (P3–5,P7–9 and P12–P13). ArcGIS 10 (ESRI 2010) was used to generate estimates for P10 and P11.

There were no estimates available in Eurostat for in- and outbound tourism from Georgia, Armenia and Azerbaijan (see P3 and P4 in Table S1). The numbers of journeys from EU MS to Georgia were found on the website of the Georgian National Tourism Agency but no data were found for Armenia or Azerbaijan, and arrivals from the three countries to EU MS were also not available.

P14, the area in each country of “suitable habitats for wild boar” was obtained through spatial analysis using the approach developed by Bosch et al. [25] based on “potential resources” i.e. suitable habitats to provide food and/or shelter to wild boars. Any such areas that did not have non-high-biosecurity farms were then removed. The area of each country with non-high-biosecurity farms was determined from data obtained through a questionnaire sent out to Chief Veterinary Officers and pig or livestock industry organisations in EU MS. The European Food Safety Authority (EFSA) definitions of high biosecurity, limited biosecurity and free range were provided [16] and respondents were asked if there were any areas of the country where only high biosecurity pigs were kept, or areas where no domestic pigs were kept. The questionnaire response rate was 67%, as no response was received for 9 countries (Estonia, Hungary, Lithuania, Luxembourg, Malta, Netherlands, Portugal, Romania and Sweden). For MS with no response, it was assumed that all areas with wild boar habitat also had pig farms with limited biosecurity.

In the questionnaire, each country was also asked what proportion of pig farms were high biosecurity, limited biosecurity or free range. Comments made by two respondents indicated that they had used farm size as a proxy for biosecurity level, and two other countries commented that they did not have data on biosecurity level and therefore the figures provided were best guesses. As a result of the uncertainty around the data for the percentage of pig farms that are non-high-biosecurity (P12), it was decided to use farm size as a proxy for biosecurity level. Indeed, previous epidemiological studies found that higher biosecurity was associated with larger herds [26]–[28].

For farm size a complete data set was available for all MS from Eurostat. Using this dataset, various farm sizes were explored based on number of breeding sows and number of pigs greater than 20 kg that were not breeding sows. The proportion of holdings with 100 or more breeding sows and/or 400 or more other pigs greater than 20 kg seemed to provide a reasonable estimate for the proportion of high biosecurity farms in a member state, when compared with the questionnaire responses that had been received. P12, the proportion of pig farms that are non-high-biosecurity, was calculated as its complementary proportion.

For some PIs the data from 2010 had a high proportion of missing values and therefore the most complete recent dataset was used (Tables S1 and S2). If only one value was available for another year then this was used, if more than one value was available for other years then the average of the nearest two values was used.

### Calculating Risk Scores

The models for release and exposure were implemented in Excel 2007 (Microsoft Corp, Redmond, WA). For each PI, natural breaks [29] were used to categorize the country values into six risk scores (RS) from 0 to 5 (Table 5). In short, natural breaks classification is a method that partition data based on natural groups in their distribution, so that between-groups variance is maximised and within-groups variance minimised.

**Table 5:** Overall risk score values and corresponding risk categories used for the release and exposure assessment.

Overall Risk score (RS) Value	Corresponding risk category
**0**	Negligible
**0<RS≤1**	Very Low
**1<RS≤2**	Low
**2<RS≤3**	Moderate
**3<RS≤4**	High
**4<RS≤5**	Very High

The relative risks of release and exposure of ASF through illegal importation of pork and pork products were then determined for each MS by weighted linear combination of the PIs’ risk scores for that country and PIs’ weights. The equations to calculate the overall release and exposure risk scores for a given MS are given below:

Overall release risk score:
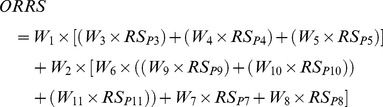
(1)and

Overall exposure risk score:
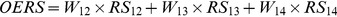
(2)where RS_Px_ is the MS risk score and W_x_ is the weight for a given proxy indicator Px (P1–P14).

The overall risk scores were converted into risk categories (Table 5) to provide for each MS the relative risk of release of ASF via illegal importation of pork and pork products, and the relative risk of exposure if ASF were released.

### Sensitivity Analysis

In addition to natural breaks, other methods of determining the ranges for risk scores were explored: dividing the range of values into 6 equal bands, dividing the values by percentiles, setting outlying values to be risk score 0 and 5 and then dividing the remaining range of values into 4 equal bands or by percentiles.

A sensitivity analysis was performed on PIs’ weights and on the number of non-high-biosecurity farms [30].

The sensitivity analysis on PIs’ weights was performed using @RISK 6 (Palisade Corporation, Newfield, NY) in Excel 2007 (Microsoft Corp, Redmond, WA). In the sensitivity analysis model for release and exposure assessment, risk scores were calculated using random combinations of pairs or triplets of individual weights given by experts, instead of calculating risk scores using the overall weight for each PI. The impact of the change in PIs’ weights on the countries’ overall risk scores was assessed after 10,000 iterations.

P12 and P14 had a high level of uncertainty because of the lack of data on farm biosecurity levels and the use of farm size as a proxy for biosecurity level. In order to examine the effect of the uncertainty of P12 and P14, fixed values for the percentage of non-high-biosecurity farms (0%–100%) were used to calculate P12 and P14, and the impact on the exposure risk category was assessed.

## Results

The weights W_1_ to W_14_ obtained from expert opinion elicitation for the release and exposure proxy indicators are shown in Figures 1 and 2.

**Figure 3:**
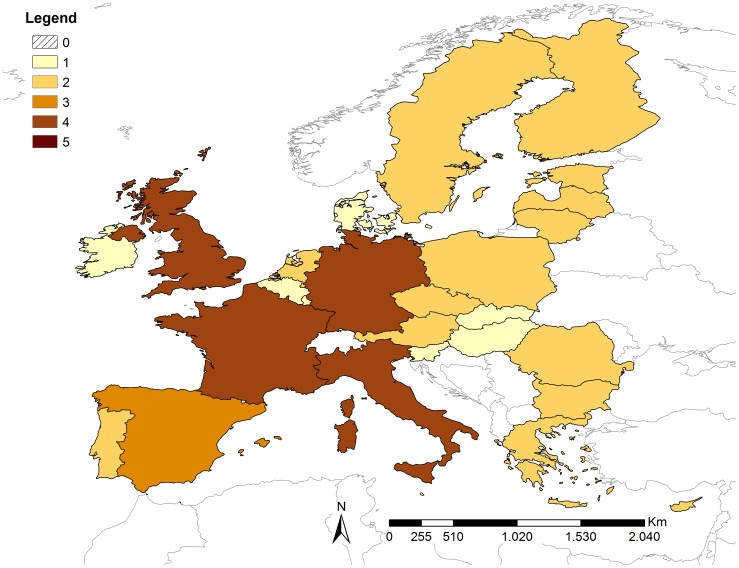
Results of the release assessment. Overall risk scores for the release of ASFV via illegal importation of pork and pork products into the European Union member states.

The overall release risk scores obtained from weighted linear combination are shown in Figure 3. No country fell into the very high risk category, but for four countries (France, Germany, Italy and United Kingdom (UK)) the risk was estimated as high and for Spain the risk of release was moderate. No country had negligible risk but eight countries had very low risk. The percentage contribution to the overall release risk score for proxy indicators P3–5 and P7–11 was based on their respective weights and equation (1). The percentage contribution was the greatest for P5/P8 (40%) because it contributed to both P1 and P2, whilst P3 (15%), P4 (13%) and P7 (16%) made lower but similar contributions. The percentage contributions of P9, P10 and P11 were 6.4%, 6.5% and 3.6%, respectively. The countries with high risk tended to have higher risk scores for outbound (P3) and inbound tourism (P4), number of residents from ASF-affected countries (P5/P8) and number of ports and airports (P9). Details of the MS risk scores for all release proxy indicators are shown in Table S3.

**Figure 4:**
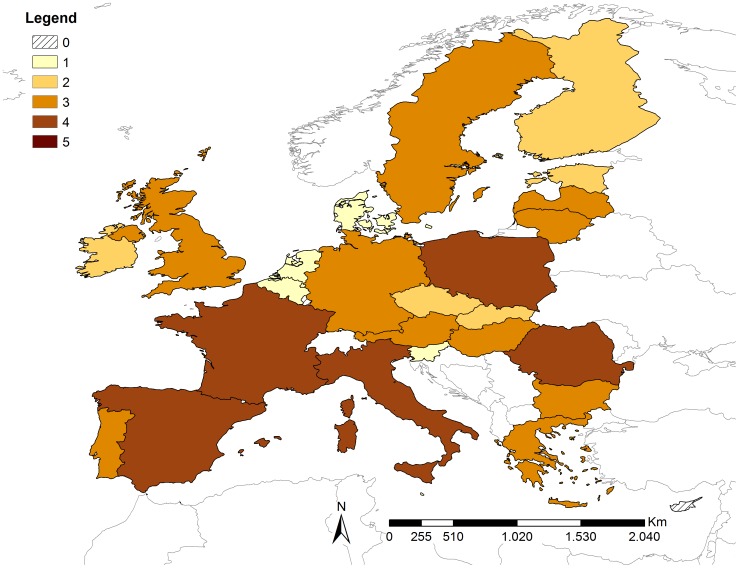
Results of the exposure assessment. Overall risk scores for the exposure of the European Union member states if ASFV was released through illegal importation of pork and pork products.

Figure 4 shows the overall exposure risk scores obtained. No country fell into the very high risk category, but five countries (France, Italy, Poland, Romania and Spain) had high risk of exposure and ten countries were in the moderate risk category (Austria, Bulgaria, Germany, Greece, Hungary, Latvia, Lithuania, Portugal, Sweden and UK). Cyprus and Luxembourg had a negligible risk, and Belgium, Denmark, Malta, The Netherlands and Slovenia had a very low risk. Romania and Poland had high risk scores for the number of non-high-biosecurity farms (5 and 4, respectively). Estonia, Portugal and Spain had high scores for the estimated number of workers in the livestock sector from ASF-affected countries (5, 5 and 4, respectively). High scores for P14, the area of country with both wild boar habitat and non-high-biosecurity farms, were obtained for France, Spain and Sweden (RS = 5), and Finland, Germany, Italy, Poland, Romania and UK (RS = 4). Details of the MS risk scores for all exposure proxy indicators are shown in Table S4.

**Figure 5:**
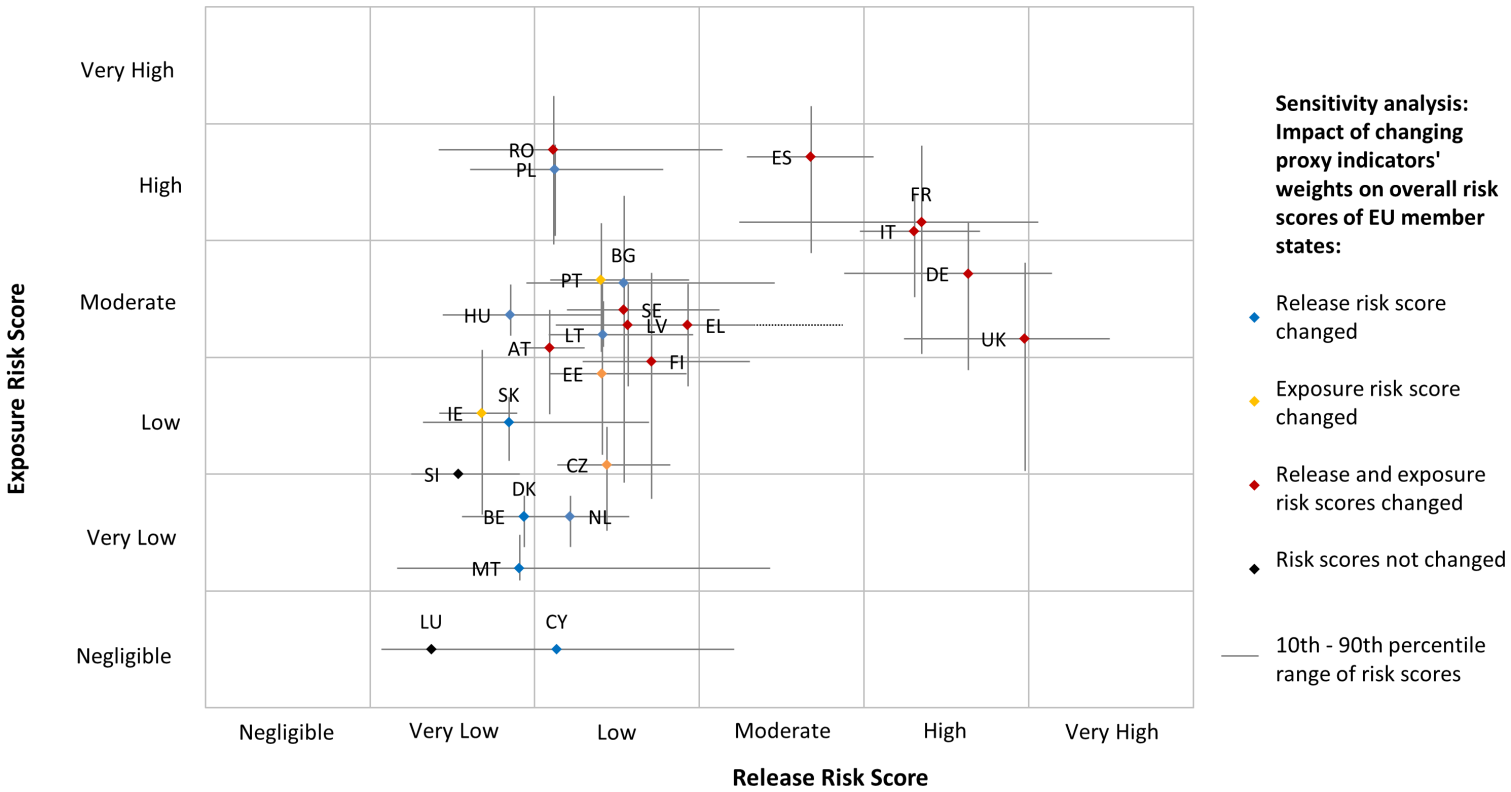
Combined results of release and exposure assessments, and results of the sensitivity analysis on proxy indicators’ weights. Scatter plot of the overall release and exposure risk scores for the European Union member states, and the 80% central interquartile range of risk scores resulting from varying proxy indicators’ weights. Abbreviations – AT: Austria, BE: Belgium, BG: Bulgaria, CY: Cyprus, CZ: Czech Republic, DK: Denmark, EE: Estonia, FI: Finland, FR: France, DE: Germany, EL: Greece, HU: Hungary, IE: Ireland, IT: Italy, LV: Latvia, LT: Lithuania, LU: Luxembourg, MT: Malta, NL: Netherlands, PL: Poland, PT: Portugal, RO: Romania, SK: Slovakia, SI: Slovenia, ES: Spain, SE: Sweden, UK: United Kingdom.

**Figure 6:**
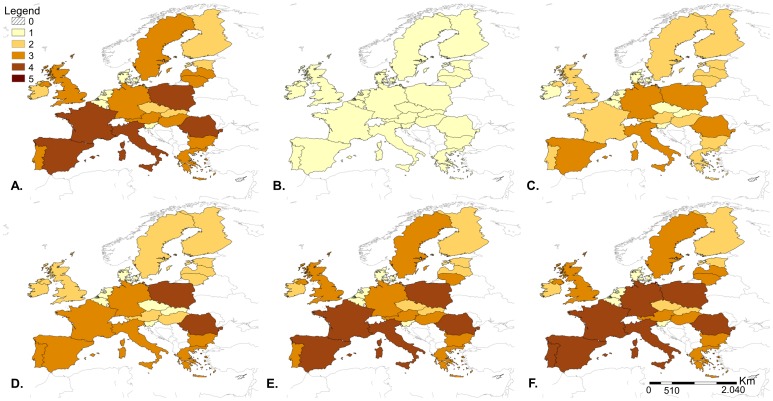
Results of the sensitivity analysis on proxy indicator P12 and P14. The overall exposure risk scores of the European Union member states were assessed for different values of percentage of non-high-biosecurity farms. Figure 6 shows the results of the exposure assessment for: a) original model, b) 0% non-high biosecurity farms, c) 25% non-high biosecurity farms, d) 50% non-high biosecurity farms, e) 75% non-high biosecurity farms, f) 100% non-high biosecurity farms. The percentage of non-high-biosecurity farms was used to calculate two exposure risk scores: RSP12 and RSP14.

Figure 5 displays the combined overall release and exposure risk scores of all EU MS, and their central 80% inter-percentile range resulting from variation in the proxy indicators’ weights. Two groups of countries have distinctively higher risk profiles: France, Germany, Italy, Spain and the United Kingdom had moderate to high risk scores for both release and exposure assessments, and Poland and Romania had low release risk scores but high exposure risk scores. Varying the PIs’ weights resulted in changes of risk of release and/or exposure for most MS, however the risk usually changed by one category only and for a small proportion of iterations (less than 25%), meaning that in the majority of cases MS’ relative risks of release and exposure remained the same. The release risk category increased within the central 50% inter-percentile range for 7 MS (Belgium, Denmark, Greece, Hungary, Malta, Slovakia and the United Kingdom), while it decreased for Cyprus, France, Poland and Romania. MS whose change in exposure risk categories occurred within the central 50% inter-percentile range are: the Czech Republic, Estonia and Finland, France, Germany, Ireland, Italy and the United Kingdom. Details of the association between PIs’ weights values and changes in MS risk categories are shown in Tables S5, S6, and S7. Some PIs’ weights had little effect on the MS relative risks, affecting few MS risk categories and only at extreme values, such as w_3_, w_6_, w_8_ and w_11_. The PIs’ weights w_2_, w_12_ and w_13_ only had an impact on risk scores at extreme values, but in that case they affected many MS risk categories. Finally, the following PIs’ weights were found to affect some MS risk categories even for values close to the mean of the individual weights given by experts: w_1_, w_4_, w_5_, w_7_, w_9_, w_10_ and w_14_. The impact of the percentage of farms with non-high-biosecurity used to calculate P12 and P14 on the overall exposure risk category is shown in Figure 6. If there were no non-high-biosecurity farms (0%) then all MS fell into the negligible or very low risk category. If 50% of farms were non-high-biosecurity, then the following countries moved down one risk category compared to the original model: Austria, Czech Republic, France, Hungary, Italy, Latvia, Lithuania, Slovakia, Spain, Sweden and UK. If 75% of farms were non-high-biosecurity, then the results were the same as the original model except that Latvia moved to the low risk category. If all farms were non-high-biosecurity (100%) then again the results were the same as the original model except that Germany and Portugal moved from moderate to high risk.

## Discussion

The semi-quantitative risk assessment model presented in this paper constitutes an innovative approach for estimating the relative risks of introduction of disease into countries through illegal importation of meat and products. The framework developed is based on a systematic and transparent approach, and makes use of available data for the 27 EU countries on factors likely to influence this risk. The results provide an indication of the likely level of the risk of introduction for countries considered, as well as the main factors contributing to it. The visual representation of the release and exposure assessments further facilitates the interpretation of the results. Another critical advantage of the approach is the use of the same framework and sources of data for all countries, allowing comparison of levels of risk between them. The model presented in this paper was developed for ASF but the approach used can easily be adapted to other diseases and other animal products.

Previous risk assessments investigating illegal importation of meat and products were informed by expert opinion for both their model structure and parameterisation, and the quantification of illegal imports relied on data on custom seizures in the case of country-specific studies [13]–[15]. The quantification of illegal movements of animal products using custom seizures constituted a challenge for these country-specific assessments, and it became a major obstacle when trying to develop a risk assessment framework suitable for all MS of the EU. Besides the fact that such data are not collected consistently across MS, it was unsure whether use of custom seizure data would have been allowed by all customs authorities. A preliminary search conducted for a small number of MS indicated that the protocols for search at customs and the data recorded varied between countries, which would have hampered comparisons between MS. Hence, a sensible compromise was to estimate the importance of illegal imports indirectly via factors likely to influence it (PIs), and for which data was available at the EU level.

This work attempted to include the main socio-economic and geographical factors known - or assumed – to influence illegal importation of meat and products. Illegal imports of meat and derived products occur either for personal consumption or for commercial purposes, i.e. for re-sale after importation [15]. Data on residents from ASF-affected countries was included to represent potential demand for animal products from these areas, which influence both the risk of smuggling for personal consumption or for commercial purposes. This is supported by a study conducted on agricultural smuggling in the United States of America (USA) which found important illegal meat imports linked to the two main foreign communities of USA immigrants: smuggling for personal consumption of specialty meats from Mexico prior to holidays, and smuggling from China for sales in ethnic food markets [31]. Tourism data to and from ASF-affected countries aimed at capturing risky small-scale imports for personal consumption by tourists carrying ASFV contaminated meat products in their luggage. While a large proportion of such tourists may be unaware of doing something illegal when importing meat products, some people know the rules but still try to bring some animal products (for example if they are considered a delicacy) into the country. Information on banned and restricted goods is usually available on posters at airports, ports and border inspection points. However, a report from the European Commission [32] indicated that little information is provided to travellers by international transport operators and that a minority of MS offer information to travellers on specific websites. Hence, passengers may not always have easy access to such information, or may find it difficult to understand. For example, an official MS webpage accessed in January 2012 [33] indicated that if travelling from another EU country it is allowed to bring in “any meat, dairy or other animal products as long as they are free from diseases and for your own consumption”. Most passengers are probably unaware of problematic diseases and how to know if the products they are carrying are free of them. Some may consider that if the products they carry are at risk they would have been informed when purchasing them or passengers may also be puzzled/misguided by products sold in some airports and duty-free areas, although banned from bringing to many regions. The EU is aware of the risk caused by personal imports and the importance of communication to the public to mitigate it, and has recently developed clearer rules for the public and produced communication packages in 35 languages (Commission Regulation (EC) No 206/2009) [34].

For the risk of illegal imports for commercial purpose, other critical factors had to be accounted for. Smuggling needs to be profitable and illegal imports usually concern animal products that are expensive and/or difficult to find. This is illustrated by the case of Norway, where high meat prices led to mass smuggling of animal products, including pork [35]. In the risk assessment model, the price of pork in each MS was used as a proxy for this economic incentive: the higher the price in a MS, the more likely the sales outweigh the cost of smuggling pork and pork products. Illegal imports for commercial purposes are also conditional on practical considerations: the easier it is to smuggle the products into a country, the more likely it is to happen. In the case of illegal imports of ASF-contaminated meat and products, the data used to capture these constraints were the distance between EU MS and ASF affected areas, and the number of entry points into a MS.

It was not possible to include data on custom seizures in the model, but our findings were compared with those of a report from the EU on illegal consignments of meat found in personal luggage at EU entry points [32]. The report showed an increase in the reported amounts of meat seizures in personal luggage between 2005 and 2007, with more than 200 tonnes confiscated and/or destroyed in 2007. This might reflect an enhancement of search methods at customs, but the figures also confirm the risk of introduction of diseases into EU posed by illegal imports of animal products. Between 2005 and 2007, the countries amongst the five most frequently cited as the origin of seized personal consignments of meat were China, Egypt, Russia, Turkey (for the 3 years), Ukraine (2006 and 2007), Romania (2005), Thailand (2006) and Serbia Montenegro (2007). Given the occurrence of ASF in Russia and the first outbreak of ASF reported in Ukraine at the end of July 2012, these findings highlight the importance of the results of the present risk assessment. The results are also in agreement with the conclusions of previous studies [16]–[19] that called attention to the importance of illegal importation of pork and products in the risk of introduction of ASF into the EU.

The EU report on illegal consignments [32] identified Spain, UK, Germany and France as the countries with the largest amounts of meat confiscated/destroyed from personal luggage, while Italy was amongst the MS with intermediate reported amounts of meat seizures in personal luggage. The results of the release risk assessment are in agreement with the EU report, as the 5 MS with moderate or high relative risks of release correspond to countries reporting large amounts of meat seizures. Differences in protocols for detection of illegal importation and/or in data reported to the EU might have contributed to the observed difference of ranking between the EU report and the model results for Italy and Spain. Also, the EU report only included illegal consignments in personal luggage and the observed difference might be explained at least partially by illegal imports for commercial purposes. Indeed, Italy had higher scores than Spain for all PIs influencing the risk of release via importations of pork and pork products for commercial purposes.

Freedom of trade within the EU means that it is possible for illegal animal products to enter through one MS and then be transported to another, most likely for commercial reasons than personal consumption. A possible scenario is that the animal product is imported to a MS for geographical reasons (e.g. proximity to an infected country, many ports and airports or less controlled border points with non-EU countries) in order to then be transported within the EU to meet high demand in a nearby MS (e.g. countries where the animal products of interest are very expensive). This scenario has not explicitly been addressed in our model. However, the results provide an indication of whether and how this could happen. In the case of ASF introduction through illegal imports of pork and products, the countries that could be initial points of introduction are those with high or very high scores on geographical parameters (P9–P11): Estonia, Finland, France, Germany, Greece, Italy, Latvia, Lithuania, Poland, Romania, Sweden and UK. No specific MS had high RS for the combined demand parameters ‘number of residents from ASF-affected countries’ and ‘price of pork’. A spatio-temporal model for ASF spread in EU recently developed also provides insights on this issue [36].

A majority of MS had high or moderate relative risks of exposure of the domestic pig population if ASF were released. These MS had a moderate to very high risk score for the area of country with both wild boar habitat and non-high-biosecurity (P14), which was the PI with the highest contribution to the exposure risk score. P14 assumes that in areas with non-high-biosecurity farms and wild boar, contact can occur between pigs and wild boar, resulting in exposure to ASF if wild boar were to become infected with ASF or act as fomites. Unless the questionnaire respondents had indicated complete absence of pigs or only high biosecurity pigs in specified areas then it was assumed that all areas with wild boar habitat also had non-high-biosecurity pigs. As only Finland, Germany and Denmark reported areas with no non-high-biosecurity farms, this may overall have resulted in an overestimation of the risk. Also, this resulted in MS risk scores for P14 being mainly driven by the area of the country with wild boar habitat suitability, hence the high risk scores for MS with large areas of potential wild boar habitat. The wild boar habitat suitability was derived from the first analysis of the distribution of the wild boar in the Iberian Peninsula [25]: the same methodology was applied across the EU to generate a distribution map allowing comparison between countries. The lack of detailed national data on both wild boar and non-high-biosecurity farms however makes more precise estimation of the areas of interface between non-high-biosecurity farms and wild boar difficult, and represents an important limitation of this study.

The five countries assessed at highest risk of exposure had different profiles: the risk for France and Italy was mainly due to the countries’ large areas of wild boar habitat. In addition to the large area of the countries with both non-high-biosecurity farms and wild boar habitat, Romania and Poland had a very large proportion of pig farms falling in the non-high-biosecurity category (i.e. holdings with less than 100 breeding sows and/or less than 400 other pigs greater than 20 kg) and hence at higher risk of swill feeding, contact with fomites and access to waste or landfill, all known to be mechanisms of transmission of ASF. In addition to large areas with wild boar habitat, Spain had a large estimated number of pig farm workers from ASF-affected countries (P13). P13 assumes that foreign pig farm workers are more likely to consume meat products from their countries of origin, imported illegally, that these products may be contaminated with ASF, and that farm workers can infect pigs either by acting as fomites or by feeding swill to pigs. In the case of Spain, pig farmers are likely to be aware of the risk of ASF introduction and thus swill feeding is less likely to occur compared to other countries with no history of ASF. While this model clearly highlights areas that are potentially of concern, country specific risk assessments are needed to confirm or clarify threats identified in the generic model.

As with any model, the results of the present risk assessment were influenced by the quality of the input data. Good data for all MS were available for most of the PIs included in the model. However, the data available and/or their quality were limited for some PIs of the exposure assessment, such as the number of non-high-biosecurity farms. A consultation of Chief Veterinary Officers from a number of MS indicated that the different levels of biosecurity as defined by EFSA [16] were interpreted differently, which prevented the use of questionnaire data for this PI. Instead farm size was used as a proxy for the level of biosecurity and the generated estimates were associated with a high level of uncertainty, as they did not account for differences in pig farming systems between MS and the fact that farm size and biosecurity level are not directly correlated [28]. The sensitivity analysis performed was useful to understand the impact of this PI on the overall exposure RS. Thanks to the systematic structure of the approach used, re-assessing the risk of introduction of ASF into EU MS through illegal importation of pork and products is easily possible as soon as more data becomes available for the model parameterisation. If ASF were to further spread and persist in Ukraine, the model parameters could be updated to account for the country status. The following data would have to be added to the current model: outbound and inbound tourism to Ukraine (data on the Ukrainian official statistics website), residents of Ukraine in MS (data in Eurostat), and distance to nearest ASF-infected countries (Poland, Slovakia, Hungary and Romania would then have very high risk score for this PI).

The sensitivity analysis carried out on on parameters with high uncertainty showed their impact on the risk assessment, and the sensitivity analysis performed on PIs’ weights provided a measure of the uncertainty in the model outputs associated to the uncertainty in the relative importance of the PIs. Overall the sensitivity analysis suggested that the model is reasonably robust as no major changes of risk levels were observed and most changes in risk categories occurred for extreme values of PIs’ weights. Model validation is also important, although difficult to perform as no estimates of the magnitude of meat smuggling into the EU were available. The previous comparison of the MS release risks with the reported seizures of meat products in personal luggage at EU entry points [34] suggested that the model performed well, identifying the same MS at higher risk. In addition, this risk assessment was compared with studies investigating ASF introduction into Finland [37], UK [15], [38], Poland [39] and Denmark [40]. Quinn [39] estimated the risk of ASF release via illegal imports to be medium for Poland, while the overall release score obtained in this study was low. Quinn considered the existence of an illegal market for pork products from ASF-infected areas to be highly unlikely, which agrees with the present risk assessment. However, Quinn considered the probability of release due to legal or illegal immigrants from Russia and Transcaucasia carrying pork products for personal consumption to be medium, whilst the current risk assessment estimated the risk for importation for personal consumption to be very low. A study on smuggling at the Polish-Ukrainian border suggests that meat smuggling is probably limited and is more likely to be from Poland to Ukraine [41], and thus supports the findings of the present study. In the risk assessment by Brown [40] the probability of release of ASF into Denmark via illegal pork imports was estimated to be negligible based on previous findings [13], while it was estimated to be very low in the current study. In this work, the risk scores for Denmark were negligible or very low for all parameters except ‘distance to nearest ASF-infected country’ and ‘number of ports and airports’ for which medium risk scores were obtained. In a recent qualitative risk assessment for the risk of introduction of ASF into the UK domestic population from endemic regions during 2012, Brand [38] estimated the risk of release via illegal imports to be moderate on a scale of four risk categories (negligible, low, moderate, high). This compares well with the current release assessment in which UK had a high release risk score. In contrast, a quantitative risk assessment for Great Britain [15] estimated a negligible or very low risk of illegal imports of ASF-contaminated meat (46 g, range = 7–138 g). However, Wooldridge’s assessment pre-dated the introduction of ASF into Russia and Transcaucasia, so their results are not comparable with the results of this work. The aforementioned risk assessments did not meaningfully differ from the findings obtained in this study by considering factors that are most likely linked with illegal importation. This supports our risk assessment results which indicated that overall there is an important risk of introduction of ASF into the EU through illegal importation of pork and pork products.

In conclusion, this paper presented an innovative semi-quantitative risk assessment model for the introduction of diseases through illegal importation of meat and derived products. The model shows that the overall risk of ASF introduction into EU via such illegal importation is not negligible. The results stress the importance of the enforcement of the restrictions on the introduction of personal consignments of meat products at EU entry points, and of good farm biosecurity including the respect of ban on swill feeding. Risk assessment models for disease introduction into the EU would benefit from the harmonisation of data collection for compilation in databases such as Eurostat. The study also highlighted the need for a common definition of biosecurity levels and categorisation of pig farming systems accordingly across EU MS.

## Supporting Information

Table S1 **Sources of data for the release proxy indicators, assumptions and uncertainty of the data.**
(DOCX)

Table S2 **Details of exposure proxy indicators, parameters, sources of data, assumptions and uncertainty of the data.**
(DOCX)

Table S3 **Detailed results of the release assessment.** The data shows for all European Union member states the detail of the release proxy indicators’ risk scores and the overall release risk score, as well as the percentage contribution of each proxy indicator’s risk score to the overall release risk score.
(DOCX)

Table S4 **Detailed results of the exposure assessment.** The data shows for all European Union member states the detail of the exposure proxy indicators’ risk scores and the overall exposure risk score, as well as the percentage contribution of each proxy indicator’s risk score to the overall exposure risk score.
(DOCX)

Table S5 **Association between increase in country release risk score and values of proxy indicator’s weights.** The data shows the overall release risk category for all European Union member states for the original release assessment model, the risk score percentile at which an increase in risk category is observed, and the values and percentiles of proxy indicators’ weights at which the increase in risk category is observed. Increases in risk category observed in the central 50% inter-percentile range are indicated in bold.
(DOCX)

Table S6 **Association between decrease in country release risk score and values of proxy indicator’s weights.** The data shows the overall release risk category for all European Union member states for the original release assessment model, the risk score percentile at which a decrease in risk category is observed, and the values and percentiles of proxy indicators’ weights at which the decrease in risk category is observed. Decreases in risk category observed in the central 50% inter-percentile range are indicated in bold.
(DOCX)

Table S7 **Association between change in country exposure risk score and values of proxy indicator’s weights.** The data shows the overall exposure risk category for all European Union member states for the original exposure assessment model, the risk score percentile at which a change in risk category is observed, and the values and percentiles of proxy indicators’ weights at which the change in risk category is observed. Changes in risk category observed in the central 50% inter-percentile range are indicated in bold.
(DOCX)

## References

[1] FAO EMPRES TADs. Available: http://www.fao.org/ag/againfo/programmes/en/empres/diseases.asp. Accessed 10 Jul 2012.

[2] OIE (2012) World Animal Health Information Database (WAHID) - World Organisation for Animal Health (OIE). Available: http://www.oie.int/wahis_2/public/wahid.php/Wahidhome/Home. Accessed 27 Jan 2012.

[3] Pharo HJ (2002) Foot-and-mouth disease: an assessment of the risks facing New Zealand. N Z Vet J 50: 46–55. 16032210 10.1080/00480169.2002.36250

[4] Moennig V, Floegel-Niesmann G, Greiser-Wilke I (2003) Clinical signs and epidemiology of classical swine fever: a review of new knowledge. Vet J 165: 11–20. 12618065 10.1016/s1090-0233(02)00112-0

[5] Paton DJ, Greiser-Wilke I (2003) Classical swine fever–an update. Res Vet Sci 75: 169–178. 13129664 10.1016/s0034-5288(03)00076-6

[6] Sanchez-Botija C, Badiola C (1966) Presencie of the African swine pest virus in *Haematopinus suis* . Bulletin - Office International des Epizooties 66: 699–705. 6011924

[7] Lyra TMP (2006) The eradication of African swine fever in Brazil, 1978–1984. Rev Sci Tech Off Int Epizoot 25: 93–103. 16796039

[8] Simeon-Negrin RE, Frias-Lepoureau MT (2002) Eradication of African Swine Fever in Cuba (1971 and 1980). In: Morilla A, Yoon KJ, Zimmerman JJ, editors. Trends in Emerging Viral Infections of Swine. Ames: Iowa State Press. 125–131.

[9] Lubisi BA, Dwarka RM, Meenowa D, Jaumally R (2009) An investigation into the first outbreak of African swine fever in the Republic of Mauritius. Transbound Emerg Dis 56: 178–188. 19432639 10.1111/j.1865-1682.2009.01078.x

[10] Rowlands RJ, Michaud V, Heath L, Hutchings G, Oura CA, et al (2008) African swine fever virus isolate, Georgia, 2007. Emerg Infect Dis 14: 1870–1874. 19046509 10.3201/eid1412.080591 PMC2634662

[11] Beltran Alcrudo D, Lubroth J, Depner K, De La Rocque S (2008) African swine fever in the Caucasus. EMPRES Watch Apr. 2008.

[12] European Commission (2007) The new Animal Health Strategy (2007–2013): "prevention is better than cure". Available: http://ec.europa.eu/food/animal/diseases/strategy/index_en.htm. Accessed 27 Jan 2012.

[13] Bronsvoort BM, Alban L, Greiner M (2008) Quantitative assessment of the likelihood of the introduction of classical swine fever virus into the Danish swine population. Prev Vet Med 85: 226–240. 18342380 10.1016/j.prevetmed.2008.01.013

[14] Hartnett E, Adkin A, Seaman M, Cooper J, Watson E, et al (2007) A quantitative assessment of the risks from illegally imported meat contaminated with foot and mouth disease virus to Great Britain. Risk Anal 27: 187–202. 17362409 10.1111/j.1539-6924.2006.00869.x

[15] Wooldridge M, Hartnett E, Cox A, Seaman M (2006) Quantitative risk assessment case study: smuggled meats as disease vectors. Rev Sci Tech 25: 105–117. 16796040 10.20506/rst.25.1.1651

[16] EFSA (2010) Scientific Opinion on African Swine Fever. EFSA Journal 2010 8: 149.

[17] Costard S, Wieland B, de Glanville W, Jori F, Rowlands R, et al (2009) African swine fever: how can global spread be prevented? Philos Trans R Soc Lond B Biol Sci 364: 2683–2696. 19687038 10.1098/rstb.2009.0098 PMC2865084

[18] Mur L, Martinez-Lopez B, Martinez-Aviles M, Costard S, Wieland B, et al (2011) Quantitative Risk Assessment for the Introduction of African Swine Fever Virus into the European Union by Legal Import of Live Pigs. Transbound Emerg Dis. DOI 10.1111/j.1865–1682.2011.01253.x. 10.1111/j.1865-1682.2011.01253.x 21831148

[19] Wieland B, Dhollander S, Salman M, Koenen F (2011) Qualitative risk assessment in a data-scarce environment: a model to assess the impact of control measures on spread of African Swine Fever. Prev Vet Med 99: 4–14. 21292336 10.1016/j.prevetmed.2011.01.001

[20] Sanchez-Vizcaino JM, Mur L, Martinez-Lopez B (2012) African Swine Fever: An Epidemiological Update. Transbound Emerg Dis. DOI 10.1111/j.1865–1682.2011.01293.x. 10.1111/j.1865-1682.2011.01293.x 22225967

[21] Saaty T (2008) Relative measurement and its generalization in decision making: why pairwise comparisons are central in mathematics for the measurement of intangible factors the analytic hierarchy/network process. RACSAM 102: 251–318.

[22] Saaty TL (1980) The Analytic Hierarchy Process: Planning, Priority Setting, Resource Allocation. New York: McGraw Hill. 287 p.

[23] Malczewski J (1999) GIS and Multicriteria Decision Analysis. New York, Chichester: John Wiley & Sons. 408 p.

[24] European Commission (2011) Eurostat. Available: http://epp.eurostat.ec.europa.eu/portal/page/portal/eurostat/home/. Accessed 16 Sept 2012.

[25] Bosch J, Peris S, Fonseca C, Martinez M, De la Torre A, et al (2012) Distribution, abundance and density of the wild boar on the Iberian Peninsula, based on the CORINE program and hunting statistics. Folia Zool Brno 61: 138–151.

[26] Boklund A, Alban L, Mortensen S, Houe H (2004) Biosecurity in 116 Danish fattening swineherds: descriptive results and factor analysis. Prev Vet Med 66: 49–62. 15579334 10.1016/j.prevetmed.2004.08.004

[27] Boklund A, Mortensen S, Houe H (2005) Biosecurity in 121 Danish sow herds. Acta Veterinaria Scandinavica Suppl. 100: 5–14. 16429801

[28] Gardner IA, Willeberg P, Mousing J (2002) Empirical and theoretical evidence for herd size as a risk factor for swine diseases. Anim Health Res Rev 3: 43–55. 12400869 10.1079/ahrr200239

[29] Jenks GF (1967) The Data Model Concept in Statistical Mapping. International Yearbook of Cartography 7: 186–190.

[30] Frey HC, Patil SR (2002) Identification and review of sensitivity analysis methods. Risk Anal 22: 553–578. 12088234

[31] Ferrier P (2010) The economics of agricultural and wildlife smuggling. Trends Organ Crim 13: 219–230.

[32] European Commission (2007) Summary of the measures taken to enforce the rules on personal imports of meat and dairy products in Member States in 2005, 2006 and 2007, in accordance with Commission Regulation (EC) No 745/2004. Available: http://ec.europa.eu/food/animal/animalproducts/personal_imports/sum_personal_imports_2005_2007_final.pdf. Accessed 9 Jul 2012.

[33] HM Revenue and Customs Buying or bringing in goods from aborad - Banned or restricted goods. Available: http://www.hmrc.gov.uk/customs/banned-restricted.htm. Accessed 9 Jul 2012.

[34] European Commission (2009) Trade and Imports of Animal Products - Introduction of personal consignements. Available: http://ec.europa.eu/food/animal/animalproducts/personal_imports/index_en.htm. Accessed 10 Jul 2012.

[35] Shields R (2007) Smuggled meat on menu in Norway. The Independent. Available: http://www.independent.co.uk/news/world/europe/smuggled-meat-on-menu-in-norway-458615.html. Accessed 10 Jul 2012.

[36] Nigsch A, Costard S, Jones BA, Pfeiffer DU, Wieland B (2013) Stochastic spatio-temporal modelling of African swine fever spread in the European Union during the high risk period. Prev Vet Med 108: 262–275. 23419785 10.1016/j.prevetmed.2012.11.003

[37] Oravainen J, Sahlström LL (2011) Possible routes of entry into the country for African swine fever – risk profile. Finnish Food Safety Authority EVIRA.

[38] Brand C (2011) What is the risk of the introduction of African swine fever into the UK domestic pig population from endemic regions over 2012? In: MSc Control of Infectious Diseases RVC Project Report.

[39] Quinn JA (2010) Risk assessment for the introduction of ASF into Poland. In: BSc Veterinary Sciences RVC Final Year Research Project Report.

[40] Brown S (2010) Risk assessment for the introduction of ASF into Denmark. In: BSc Veterinary Sciences RVC Final Year Research Project Report.

[41] Polese A (2012) Who has the right to forbid and who to trade? Making sense of illegality on the Polish-Ukrainian border. In: Miggelbrink BBaJ, editor. Subverting Borders: Doing Research on Smuggling and Small Scale Trade. Leipzig: VS Verlag für Sozialwissenschaften. 21–38.

