# Supplementary material for: Introduction of African Swine Fever into the European Union through Illegal Importation of Pork and Pork Products
Source: PLoS One. 2013 Apr 15;8(4):e61104. doi: 10.1371/journal.pone.0061104 (PMC3627463; doi:10.1371/journal.pone.0061104)
Supplement: Table S1 — Sources of data for the release proxy indicators, assumptions and uncertainty of the data. (DOCX) [file pone.0061104.s001.docx]

**Table S1. Sources of data for the release proxy indicators, assumptions and uncertainty of the data.**

| **Proxy Indicator** | **Description** | **Source** | **Assumptions** | **Uncertainty** |
| --- | --- | --- | --- | --- |
| P3 | Outbound tourism to ASF-affected countries | Eurostat: holiday or business trips of one night or more from EU member states to Africa and Russia during 2007 (<http://appsso.eurostat.ec.europa.eu/nui/show.do?dataset=tour_dem_ttw&lang=en>) |  | Data from 2007 may not reflect current situation |
|  |  | Georgian National Tourism Agency: arrivals of non-resident visitors at national borders of Georgia by country of citizenship (<http://www.gnta.ge/upload/file/2000-2011_Arrivals_foreign_travellers_at_national_borders_of_Georgia.pdf>) |  | No data for trips to Armenia and Azerbaijan |
| P4 | Inbound tourism from ASF-affected countries | Eurostat: arrivals to EU member states of non-residents from Africa and Russia staying in hotels, guesthouses, etc. in 2007 (<http://appsso.eurostat.ec.europa.eu/nui/show.do?dataset=tour_occ_arnrmw&lang=en>) |  | Data from 2007 may not reflect the current situation; no data for arrivals from Armenia, Azerbaijan and Georgia; does not include arrivals staying in private accommodation |
| P5, P8 | Residents from ASF-affected countries | Eurostat: people who are residents of EU member states but are citizens of Russia, Armenia, Georgia, Azerbaijan and ASF-infected African countries 2009  (<http://appsso.eurostat.ec.europa.eu/nui/show.do?dataset=migr_pop1ctz&lang=en>) |  | Data from 2009 may not reflect the current situation |
|  |  | ASF disease status: OIE WAHIS Disease timelines 2005-2012 (http://web.oie.int/wahis/public.php?page=disease_timelines) | There was no OIE data on ASF for seven countries so those in north Africa (Eritrea, Mali, Mauritania) were assumed to not be infected and those in sub-Saharan Africa (Equatorial Guinea, Gambia, Guinea, Liberia) were assumed to be infected | ASF-affected African countries are defined as all countries that have ever been infected with ASF according to OIE WAHIS and Handistatus II databases |
|  |  | ASF disease status: OIE WAHIS List of countries by sanitary situation (http://web.oie.int/wahis/public.php?page=disease_status_lists) | Same as above | Same as above |
|  |  | ASF disease status: Handistatus II (http://web.oie.int/hs2/report.asp?lang=en 1996-2004) | Same as above | Same as above |
| P7 | Price of pork | European community: 2011 annual average price of Grade E carcasses (55-59% lean meat percentage) in Euro per 100kg (<http://ec.europa.eu/agriculture/markets/pig/porcs.pdf>) | Price of other pig products has similar variation between countries | Data from 2011 may not reflect current situation |
| P9 | Number of ports and airports | World Port Index 2009: major ports and terminals  (<http://msi.nga.mil/NGAPortal/MSI.portal?_nfpb=true&_pageLabel=msi_portal_page_62&pubCode=0015>) | The number of major ports is proportional to the total number of ports for each EU member state | Data from 2009 may not reflect the current situation |
|  |  | Eurostat: number of airports with more than 15,000 passenger movement per year (http://appsso.eurostat.ec.europa.eu/nui/show.do?dataset=avia_if_arp&lang=en) | The number of larger airports is proportional to the total number of airports for each EU member state | Data from 2008 may not reflect the current situation |
| P10 | Distance to nearest ASF-affected country | Shapefile of national boundaries: shortest distance in km from EU state border to nearest ASF-affected country. |  |  |
|  |  | ASF status of African countries (http://web.oie.int/wahis/public.php?page=home) | Only considered affected countries outside the EU, not Sardinia; in Africa the nearest ASF-affected countries based on the OIE WAHIS database were Senegal, Burkina Faso, Niger and Chad |  |
| P11 | Number of international border points with non-EU member states | FAO Geonetwork: shapefiles of railways (RWDB2 railway Lines), roads (Roads of the World VNAP0) and waterways (Perennial Water Courses (Rivers) of the World VMAP0) (<http://www.fao.org/geonetwork/srv/en/main.home>) were downloaded and overlaid on national boundary shapefile; counts of railway lines, perennial water courses and road crossing national boundaries with non-EU member states were created | Borders with all non-EU member states were included except members of the European Free Trade Association (EFTA - Switzerland, Lichtenstein, Norway –see <http://www.efta.int/>) | Navigability of water courses is not known |
