# Supplementary material for: Introduction of African Swine Fever into the European Union through Illegal Importation of Pork and Pork Products
Source: PLoS One. 2013 Apr 15;8(4):e61104. doi: 10.1371/journal.pone.0061104 (PMC3627463; doi:10.1371/journal.pone.0061104)
Supplement: Table S2 — Details of exposure proxy indicators, parameters, sources of data, assumptions and uncertainty of the data. (DOCX) [file pone.0061104.s002.docx]

## Table S2. Details of exposure proxy indicators, parameters, sources of data, assumptions and uncertainty of the data.

| **Proxy Indicator** | **Description** | **Source** | **Assumptions** | **Uncertainty** |
| --- | --- | --- | --- | --- |
| P12 | Number of pig farms that are not high biosecurity | Eurostat: number of holdings with 400+ other pigs (>20kg not breeding sows) (<http://appsso.eurostat.ec.europa.eu/nui/show.do?dataset=ef_ls_gvopig&lang=en>); number of holdings with 100+ breeding sows (<http://appsso.eurostat.ec.europa.eu/nui/show.do?dataset=ef_ls_gvsows&lang=en>); holdings with both 100+ breeding sows and 400+ other pigs obtained on request | Assumes that farms with larger numbers of breeding sows and/or fatteners are likely to have higher biosecurity measures than smaller farms, uses 100+ breeding sows and/or 400+ fatteners as a proxy for higher biosecurity level | Data from 2007 may not reflect the current situation; the use of farm size as a proxy for biosecurity has a high level of uncertainty |
| P13 | Number of pig farm workers from ASF-affected countries | P13 = (P13a/P13b)(P13c/P13b) or [(Number of livestock workers)/(National population)] x [(Number of residents who are citizens of ASF-affected countries)/(National population)] | Assumes that the proportion of pig farm workers who are citizens of ASF-affected countries is the same as the proportion of the total population who are citizens of ASF-affected countries |  |
| P13a | Number of livestock workers | Eurostat: number of persons in regular labour force (family and non-family) working on the following farm types: specialist granivores, mixed livestock mainly grazing, mixed livestock mainly granivores, various crops and livestock combined (<http://appsso.eurostat.ec.europa.eu/nui/show.do?dataset=ef_ov_lfft&lang=en>) | Assumes that all of the types of farm considered have one or more pigs | Data from 2007 may not reflect the current situation; does not include casual labour; included all types of farms that might have pigs but some may not have pigs |
| P13b | National population | Eurostat (<http://appsso.eurostat.ec.europa.eu/nui/show.do?dataset=demo_pjan&lang=en>) |  | Data from 2007 used as denominator for livestock workers and data from 2008 used as denominator for residents of ASF-  affected countries |
| P13c | Residents who are citizens of ASF-affected countries | Eurostat - 2009 population by citizenship (people usually resident) - most complete data set (<http://appsso.eurostat.ec.europa.eu/nui/show.do?dataset=migr_pop1ctz&lang=en>) |  | Data from 2009 is the most complete recent dataset but ay not reflect current situation |
|  |  | ASF disease status: OIE WAHIS Disease timelines 2005-2012 (http://web.oie.int/wahis/public.php?page=disease_timelines) | There was no OIE data on ASF for seven countries so those in north Africa (Eritrea, Mali, Mauritania) were assumed to not be infected and those in sub-Saharan Africa (Equatorial Guinea, Gambia, Guinea, Liberia) were assumed to be infected | ASF-affected African countries are defined as all countries that have ever been infected with ASF according to OIE WAHIS and Handistatus II databases |
|  |  | ASF disease status: OIE WAHIS List of countries by sanitary situation (http://web.oie.int/wahis/public.php?page=disease_status_lists) | Same as above | Same as above |
|  |  | ASF disease status: Handistatus II (http://web.oie.int/hs2/report.asp?lang=en 1996-2004) | Same as above | Same as above |
| P14 | Area of country with both wild boar habitat and non-high-biosecurity farms | (Area of country with wild boar habitat) -(Areas of country without low biosecurity farms) |  | Does not take into account either density of wild boar population or density of low biosecurity pig farms |
| P14a | Area of country with wild boar habitat | Spatial analysis by Centro de Investigación en Sanidad Animal, INIA | Where there is wild boar habitat then wild boat may be present | Wild boar habitat does not necessarily indicate number or density of wild boar population |
| P14b | Area of country without non-high-biosecurity farms | Questionnaire sent to CVOs March 2011, for countries not responding - examined Eurostat data of number of pig farms by region to identify areas with no pigs (http://appsso.eurostat.ec.europa.eu/nui/show.do?dataset=ef_olsaareg&lang=en) | Areas of the country that have no pigs at all and areas that have only high biosecurity pigs | Information provided by questionnaire respondents may not be accurate |
