# Supplementary material for: Introduction of African Swine Fever into the European Union through Illegal Importation of Pork and Pork Products
Source: PLoS One. 2013 Apr 15;8(4):e61104. doi: 10.1371/journal.pone.0061104 (PMC3627463; doi:10.1371/journal.pone.0061104)
Supplement: Table S3 — Detailed results of the release assessment. The data shows for all European Union member states the detail of the release proxy indicators’ risk scores and the overall release risk score, as well as the percentage contribution of each proxy indicator’s risk score to the overall release risk score. (DOCX) [file pone.0061104.s003.docx]

### Table S3. Detailed results of the release assessment.

| **Country** | **RS_P3_** | **RS_P4_** | **RS_P5/8_** | **RS_P7_** | **RS_P9_** | **RS_P10_** | **RS_P11_** | **Overall release risk score** | **Risk category** |
| --- | --- | --- | --- | --- | --- | --- | --- | --- | --- |
| Austria | 1 | 2 | 1 | 1 | 0 | 2 | 0 | 1.1 | low |
| Belgium | 1 | 2 | 1 | 0 | 1 | 1 | 0 | 0.9 | very low |
| Bulgaria | 0 | 2 | 1 | 4 | 0 | 3 | 2 | 1.5 | low |
| Cyprus | 0 | 2 | 0 | 4 | 1 | 3 | 0 | 1.1 | low |
| Czech R. | 1 | 3 | 1 | 2 | 0 | 3 | 0 | 1.4 | low |
| Denmark | 1 | 0 | 1 | 0 | 3 | 3 | 0 | 0.9 | very low |
| Estonia | 1 | 1 | 1 | 2 | 1 | 5 | 1 | 1.4 | low |
| Finland | 1 | 3 | 1 | 1 | 3 | 5 | 3 | 1.7 | low |
| France | 5 | 4 | 4 | 1 | 4 | 1 | 0 | 3.3 | high |
| Germany | 5 | 4 | 4 | 2 | 4 | 3 | 0 | 3.6 | high |
| Greece | 0 | 3 | 1 | 4 | 4 | 3 | 2 | 1.9 | low |
| Hungary | 1 | 1 | 0 | 2 | 0 | 3 | 2 | 0.9 | very low |
| Ireland | 1 | 0 | 1 | 0 | 2 | 0 | 0 | 0.7 | very low |
| Italy | 3 | 5 | 3 | 4 | 4 | 2 | 0 | 3.3 | high |
| Latvia | 1 | 1 | 1 | 3 | 1 | 5 | 1 | 1.6 | low |
| Lithuania | 1 | 1 | 1 | 2 | 0 | 5 | 3 | 1.4 | low |
| Luxembourg | 0 | 0 | 0 | 2 | 0 | 1 | 0 | 0.4 | very low |
| Malta | 0 | 0 | 0 | 5 | 0 | 2 | 0 | 0.9 | very low |
| Netherlands | 2 | 2 | 1 | 0 | 2 | 2 | 0 | 1.2 | low |
| Poland | 1 | 2 | 0 | 1 | 2 | 4 | 5 | 1.1 | low |
| Portugal | 1 | 2 | 1 | 3 | 2 | 0 | 0 | 1.4 | low |
| Romania | 1 | 0 | 0 | 3 | 2 | 3 | 5 | 1.1 | low |
| Slovakia | 1 | 0 | 0 | 3 | 0 | 3 | 1 | 0.9 | very low |
| Slovenia | 1 | 0 | 0 | 1 | 0 | 2 | 3 | 0.5 | very low |
| Spain | 2 | 4 | 3 | 3 | 3 | 0 | 0 | 2.7 | moderate |
| Sweden | 1 | 2 | 1 | 1 | 4 | 5 | 0 | 1.5 | low |
| United Kingdom | 4 | 4 | 5 | 3 | 5 | 1 | 0 | 4.0 | high |
| **Contribution to overall score (%)** | 15.2 | 12.7 | 39.8 | 15.8 | 6.4 | 6.5 | 3.6 |  |  |

The data shows for all European Union member states the detail of the release proxy indicators’ risk scores and the overall release risk score, as well as the percentage contribution of each proxy indicator’s risk score to the overall release risk score.
