# Supplementary material for: Introduction of African Swine Fever into the European Union through Illegal Importation of Pork and Pork Products
Source: PLoS One. 2013 Apr 15;8(4):e61104. doi: 10.1371/journal.pone.0061104 (PMC3627463; doi:10.1371/journal.pone.0061104)
Supplement: Table S5 — Association between increase in country release risk score and values of proxy indicator’s weights. The data shows the overall release risk category for all European Union member states for the original release assessment model, the risk score percentile at which an increase in risk category is observed, and the values and percentiles of proxy indicators’ weights at which the increase in risk category is observed. Increases in risk category observed in the central 50% inter-percentile range are indicated in bold. (DOCX) [file pone.0061104.s005.docx]

### Table S5. Association between increase in country release risk score and values of proxy indicator’s weights.

| **Country** | **Release risk category** | **Risk score percentile** | **W_1_ values (percentile)** | **W_2_ values (percentile)** | **W_3_ values (percentile)** | **W_4_ values (percentile)** | **W_5_ values (percentile)** | **W_6_ values (percentile)** | **W_7_ values (percentile)** | **W_8_ values (percentile)** | **W_9_ values (percentile)** | **W_10_ values (percentile)** | **W_11_ values (percentile)** |
| --- | --- | --- | --- | --- | --- | --- | --- | --- | --- | --- | --- | --- | --- |
| **Austria** | low | n/a | n/a | n/a | n/a | n/a | n/a | n/a | n/a | n/a | n/a | n/a | n/a |
| **Belgium** | very low | **56^th^** | n/a | n/a | n/a | n/a | <0.44(42^nd^ | n/a | n/a | n/a | n/a | n/a | n/a |
| **Bulgaria** | low | 80^th^ | <0.25(38^th^) | >0.75(85^th^) | n/a | n/a | n/a | n/a | >0.62(78^th^) | n/a | n/a | >0.57(85^th^) | n/a |
| **Cyprus** | low | 86^th^ | <0.25(38^th^) | >0.75(85^th^) | n/a | n/a | n/a | n/a | >0.63(87^th^) | n/a | n/a | n/a | n/a |
| **Czech R.** | low | 97^th^ | n/a | n/a | n/a | >0.62(99^th^) | <0.14 (8^th^) | n/a | n/a | n/a | n/a | >0.57(85^th^) | n/a |
| **Denmark** | very low | **67^th^** | <0.25(38^th^) | >0.75(85^th^) | n/a | n/a | n/a | >0.57(82^nd^ | n/a | n/a | n/a | n/a | n/a |
| **Estonia** | low | 91^st^ | <0.17(15^th^) | >0.83(96^th^) | n/a | n/a | n/a | >0.57(82^nd^ | n/a | n/a | <0.2 (26^th^) | >0.57(85^th^) | n/a |
| **Finland** | low | 79^th^ | <0.25(38^th^) | >0.75(85^th^) | n/a | n/a | <0.41(39^th^) | >0.57(82^nd^ | n/a | n/a | n/a | n/a | n/a |
| **France** | high | 86^th^ | n/a | n/a | >0.57(89^th^) | n/a | <0.29(28^th^) | n/a | n/a | >0.45(78^th^) | >0.48(66^th^) | n/a | n/a |
| **Germany** | high | 75^th^ | n/a | n/a | >0.57(89^th^) | n/a | <0.30(31^st^) | n/a | n/a | n/a | n/a | n/a | n/a |
| **Greece** | low | **59^th^** | <0.25(38^th^) | >0.75(85^th^) | n/a | n/a | <0.47(46^th^) | n/a | n/a | n/a | n/a | n/a | n/a |
| **Hungary** | very low | **69^th^** | <0.25(38^th^) | >0.75(85^th^) | n/a | n/a | <0.44(42^nd^ | n/a | >0.57(72^nd^ | n/a | n/a | >0.57(85^th^) | n/a |
| **Ireland** | very low | 99^th^ | <0.17(15^th^) | >0.83(96^th^) | n/a | <0.10(27^th^) | n/a | >0.63(86^th^) | <0.14(22^nd^ | n/a | >0.66(99^th^) | <0.25(35^th^) | 0.09(10^th^) |
| **Italy** | high | 98^th^ | n/a | n/a | n/a | >0.62(99^th^) | <0.14(8^th^) | n/a | >0.57(72^nd^ | n/a | >0.45(61^st^) | n/a | n/a |
| **Latvia** | low | 78^th^ | <0.25(38^th^) | >0.75(85^th^) | n/a | n/a | n/a | n/a | >0.57(72^nd^ | n/a | n/a | >0.57(85^th^) | n/a |
| **Lithuania** | low | 90^th^ | <0.25(38^th^) | >0.75(85^th^) | n/a | n/a | n/a | >0.57(82^nd^ | n/a | n/a | <0.14(21^st^) | >0.5 (85^th^) | n/a |
| **LX*** | very low | 90^th^ | <0.17(15^th^) | >0.83(96^th^) | n/a | n/a | n/a | n/a | >0.67(91^st^) | n/a | n/a | n/a | n/a |
| **Malta** | very low | **70^th^** | <0.25(38^th^) | >0.75(85^th^) | n/a | n/a | n/a | n/a | >0.54(68^th^) | n/a | n/a | n/a | n/a |
| **NL**** | low | n/a | n/a | n/a | n/a | n/a | n/a | n/a | n/a | n/a | n/a | n/a | n/a |
| **Poland** | low | 93^rd^ | <0.25(38^th^) | >0.75(85^th^) | n/a | n/a | <0.47(46^th^) | >0.66(96^th^) | n/a | n/a | <0.2(26^th^) | >0.57(85^th^) | n/a |
| **Portugal** | low | 92^nd^ | <0.17(15^th^) | >0.83(96^th^) | n/a | n/a | <0.47(46^th^) | n/a | >0.67(91^st^) | n/a | >0.57(75^th^) | <0.29(39^th^) | n/a |
| **Romania** | low | 83^rd^ | <0.17(15^th^) | >0.83(96^th^) | n/a | n/a | n/a | n/a | >0.62(78^th^) | n/a | n/a | n/a | n/a |
| **Slovakia** | very low | **69^th^** | <0.25(38^th^) | >0.75(85^th^) | n/a | n/a | <0.47(46^th^) | n/a | >0.57(72^nd^ | n/a | n/a | >0.57(85^th^) | n/a |
| **Slovenia** | very low | 93^rd^ | <0.17(15^th^) | >0.83(96^th^) | n/a | n/a | <0.41(39^th^) | >0.57(82^nd^ | n/a | n/a | <0.14(21^st^) | >0.57(85^th^) | >0.29(90^th^) |
| **Spain** | moderate | 85^th^ | n/a | n/a | <0.11(23^rd^) | >0.47(88^th^) | <0.41(39^th^) | n/a | n/a | n/a | >0.45(61^st^) | n/a | n/a |
| **Sweden** | low | 87^th^ | <0.25(38^th^) | >0.75(85^th^) | n/a | n/a | n/a | >0.63(86^th^) | n/a | n/a | n/a | >0.57(85^th^) | n/a |
| **UK***** | high | **41^st^** | n/a | n/a | n/a | n/a | n/a | n/a | n/a | n/a | n/a | n/a | n/a |

*LX: Luxembourg; **NL: Netherlands; *** UK: United Kingdom

The data shows the release risk category for all European Union member states for the original release assessment model, the risk score percentile at which an increase in risk category is observed, and the values and percentiles of proxy indicators’ weights at which the increase in risk category is observed. Increases in risk category observed in the central 50% inter-percentile range are indicated in bold.
