# Supplementary material for: Introduction of African Swine Fever into the European Union through Illegal Importation of Pork and Pork Products
Source: PLoS One. 2013 Apr 15;8(4):e61104. doi: 10.1371/journal.pone.0061104 (PMC3627463; doi:10.1371/journal.pone.0061104)
Supplement: Table S7 — Association between change in country exposure risk score and values of proxy indicator’s weights. The data shows the overall exposure risk category for all European Union member states for the original exposure assessment model, the risk score percentile at which a change in risk category is observed, and the values and percentiles of proxy indicators’ weights at which the change in risk category is observed. Changes in risk category observed in the central 50% inter-percentile range are indicated in bold. (DOCX) [file pone.0061104.s007.docx]

### Table S7. Association between change in country exposure risk score and values of proxy indicator’s weights.

|  |  | **Risk category increase by one level** | | | | **Risk category decreased by one level** | | | |
| --- | --- | --- | --- | --- | --- | --- | --- | --- | --- |
| **Country** | **Exposure risk category** | **Risk score percentile** | **W_12_ values (percentile)** | **W_13_ values (percentile)** | **W_14_ values (percentile)** | **Risk score percentile** | **W_12_ values (percentile)** | **W_13_ values (percentile)** | **W_14_ values (percentile)** |
| **Austria** | moderate | n/a | n/a | n/a | n/a | 40^th^ | >0.47 (80^th^) | >0.24 (75^th^) | <0.28 (25^th^) |
| **Belgium** | very low | n/a | n/a | n/a | n/a | n/a | n/a | n/a | n/a |
| **Bulgaria** | moderate | n/a | n/a | n/a | n/a | n/a | n/a | n/a | n/a |
| **Cyprus** | negligible | n/a | n/a | n/a | n/a | n/a | n/a | n/a | n/a |
| **Czech R.** | low | n/a | n/a | n/a | n/a | **40^th^** | >0.47 (80^th^) | >0.24 (75^th^) | <0.28 (25^th^) |
| **Denmark** | very low | n/a | n/a | n/a | n/a | n/a | n/a | n/a | n/a |
| **Estonia** | low | **65^th^** | n/a | >0.32 (85^th^) | <0.33 (40^th^) | n/a | n/a | n/a | n/a |
| **Finland** | low | **50^th^** | n/a | n/a | >0.63 (80^th^) | 15^th^ | >0.62 (95^th^) | >0.25 (80^th^) | <0.11 (10^th^) |
| **France** | high | 95^th^ | 0.14 <(10^th^) | n/a | >0.71 (100^th^) | **40^th^** | >0.47 (80^th^) | >0.25 (80^th^) | <0.21 (20^th^) |
| **Germany** | moderate | **55^th^** | <0.25 (25^th^) | n/a | >0.63 (80^th^) | 10^th^ | >0.62 (95^th^) | >0.24 (75^th^) | <0.14 (15^th^) |
| **Greece** | moderate | n/a | n/a | n/a | n/a | 20^th^ | >0.62 (95^th^) | >0.24 (75^th^) | <0.14 (15^th^) |
| **Hungary** | moderate | n/a | n/a | n/a | n/a | n/a | n/a | n/a | n/a |
| **Ireland** | low | **70^th^** | <0.25 (25^th^) | n/a | >0.66 (85^th^) | **30^th^** | >0.61 (90^th^) | >0.25 (80^th^) | <0.14 (15^th^) |
| **Italy** | high | n/a | n/a | n/a | n/a | **40^th^** | >0.47 (80^th^) | >0.47 (80^th^) | <0.21 (20^th^) |
| **Latvia** | moderate | n/a | n/a | n/a | n/a | 20^th^ | >0.62 (95th) | >0.24 (75th) | <0.14 (15th) |
| **Lithuania** | moderate | n/a | n/a | n/a | n/a | n/a | n/a | n/a | n/a |
| **Luxembourg** | negligible | n/a | n/a | n/a | n/a | n/a | n/a | n/a | n/a |
| **Malta** | very low | n/a | n/a | n/a | n/a | n/a | n/a | n/a | n/a |
| **Netherlands** | very low | n/a | n/a | n/a | n/a |  | n/a | n/a | n/a |
| **Poland** | high | n/a | n/a | n/a | n/a | 5^th^ | <0.14 (5^th^) | >0.57 (100^th^) | <0.29 (30^th^) |
| **Portugal** | moderate | 90^th^ | n/a | >0.48 (95^th^) | <0.29 (30^th^) | 5^th^ | >0.61 (90^th^) | n/a | <0.30 (35^th^) |
| **Romania** | High | 80^th^ | >0.60 (85th) | n/a | <0.11 (10^th^) | 10^th^ | n/a | >0.48 (95^th^) | <0.30 (35^th^) |
| **Slovakia** | low | n/a | n/a | n/a | n/a | n/a | n/a | n/a | n/a |
| **Slovenia** | very low | n/a | n/a | n/a | n/a | n/a | n/a | n/a | n/a |
| **Spain** | high | **55^th^** | <0.25 (25^th^) | n/a | >0.63 (80^th^) | 10^th^ | >0.62 (95^th^) | >0.24 (75^th^) | <0.14 (15^th^) |
| **Sweden** | moderate | **60^th^** | <0.25 (25^th^) | n/a | >0.66 (85^th^) | 10^th^ | >0.60 (85th) | >0.25 (80^th^) | <0.21 (20^th^) |
| **United Kingdom** | moderate | 95^th^ | 0.14 <(10^th^) | n/a | >0.71 (100^th^) | **40^th^** | >0.47 (80^th^) | >0.25 (80^th^) | <0.21 (20^th^) |

The data shows the exposure risk category for all European Union member states for the original exposure assessment model, the risk score percentile at which a change in risk category is observed, and the values and percentiles of proxy indicators’ weights at which the change in risk category is observed. Changes in risk category observed in the central 50% inter-percentile range are indicated in bold.
